# Supplementary material for: Variation in salivary cortisol responses in yearling Thoroughbred racehorses during their first year of training
Source: PLoS One. 2023 Apr 6;18(4):e0284102. doi: 10.1371/journal.pone.0284102 (PMC10079128; doi:10.1371/journal.pone.0284102)
Supplement: S1 Table — (DOCX) [file pone.0284102.s001.docx]

| **ID** | **FD_T1** | **FD_T2** | **FG_T1** | **FG_T2** | **FR_T1** | **FR_T2** | **RN** | **RY_T1** | **RY_T2** | **T1** | **T2** | **T3** | **T4** | **T5** |
| --- | --- | --- | --- | --- | --- | --- | --- | --- | --- | --- | --- | --- | --- | --- |
| 1 | 5.60 | 10.88 | NA | NA | NA | NA | NA | NA | NA | NA | NA | NA | NA | NA |
| 2 | NA | NA | NA | NA | NA | NA | 2.39 | NA | NA | NA | NA | NA | NA | NA |
| 3 | NA | NA | NA | NA | 1.89 | 13.43 | NA | 1.38 | 1.45 | NA | NA | NA | NA | NA |
| 4 | NA | NA | NA | NA | 4.42 | 3.84 | 2.33 | 1.04 | 1.64 | NA | NA | NA | NA | NA |
| 5 | NA | NA | NA | NA | 5.31 | 15.30 | NA | 4.77 | 2.31 | NA | NA | NA | NA | NA |
| 6 | NA | NA | NA | NA | 2.43 | 11.30 | NA | 2.23 | 1.74 | 2.55 | 1.82 | 2.03 | 1.70 | 1.56 |
| 7 | NA | NA | NA | NA | NA | NA | 2.94 | 1.20 | NA | NA | NA | NA | NA | NA |
| 8 | NA | NA | NA | NA | NA | NA | NA | 2.18 | 2.21 | NA | NA | NA | NA | NA |
| 9 | NA | NA | 1.93 | 4.40 | NA | NA | NA | 1.97 | 1.38 | NA | NA | NA | NA | NA |
| 10 | NA | NA | NA | NA | 5.60 | NA | 3.11 | 1.42 | 2.29 | NA | NA | NA | NA | NA |
| 11 | NA | 3.89 | 2.67 | 9.95 | NA | NA | NA | 1.54 | NA | NA | NA | NA | NA | NA |
| 12 | NA | NA | NA | NA | NA | 15.84 | 3.48 | 1.49 | 1.90 | NA | NA | NA | NA | NA |
| 13 | 2.42 | 8.37 | NA | NA | NA | NA | NA | 1.86 | 1.94 | NA | NA | NA | NA | NA |
| 14 | NA | NA | NA | NA | NA | NA | 1.82 | 3.16 | NA | NA | NA | NA | NA | NA |
| 15 | NA | NA | NA | NA | 1.74 | 6.79 | 1.28 | 1.17 | 1.52 | NA | NA | NA | NA | NA |
| 16 | NA | NA | NA | NA | NA | NA | 2.70 | 2.71 | NA | NA | NA | NA | NA | NA |
| 17 | NA | NA | NA | NA | NA | NA | 1.52 | NA | NA | NA | NA | NA | NA | NA |
| 18 | NA | NA | NA | NA | 1.53 | 3.70 | 1.66 | 1.76 | 2.10 | NA | NA | NA | NA | NA |
| 19 | NA | NA | NA | NA | NA | NA | NA | 1.89 | NA | NA | NA | NA | NA | NA |
| 20 | NA | NA | NA | NA | NA | NA | 4.34 | 1.84 | 2.13 | NA | NA | NA | NA | NA |
| 21 | NA | NA | NA | NA | NA | NA | 1.28 | NA | 3.82 | NA | NA | NA | NA | NA |
| 22 | NA | NA | NA | NA | NA | 13.13 | NA | 3.82 | 2.70 | NA | NA | NA | NA | NA |
| 23 | NA | NA | NA | NA | 2.54 | 4.01 | 3.23 | 2.30 | 1.13 | NA | NA | NA | NA | NA |
| 24 | NA | NA | NA | NA | NA | 6.41 | NA | NA | NA | NA | NA | NA | NA | NA |
| 25 | NA | NA | NA | NA | NA | 8.52 | 2.21 | 1.41 | NA | NA | NA | NA | NA | NA |
| 26 | NA | NA | NA | NA | NA | NA | 2.53 | 3.06 | NA | NA | NA | NA | NA | NA |
| 27 | NA | NA | NA | NA | NA | NA | 1.81 | NA | NA | NA | NA | NA | NA | NA |
| 28 | NA | NA | NA | NA | 4.24 | 12.07 | 1.88 | 2.51 | NA | NA | NA | NA | NA | NA |
| 29 | NA | NA | 3.85 | 14.45 | NA | NA | NA | 5.24 | 1.24 | NA | NA | NA | NA | NA |
| 30 | NA | NA | NA | NA | NA | NA | 1.40 | NA | NA | NA | NA | NA | NA | NA |
| 31 | NA | NA | NA | NA | 2.85 | 12.28 | 2.38 | 2.31 | 5.81 | NA | NA | NA | NA | NA |
| 32 | NA | NA | NA | NA | NA | 9.94 | 3.50 | 3.02 | 1.03 | 4.00 | 2.44 | 1.98 | 1.59 | 2.36 |
| 33 | NA | NA | NA | NA | 3.66 | 11.59 | NA | 3.50 | 3.67 | NA | NA | NA | NA | NA |
| 34 | 3.23 | 3.88 | NA | NA | 1.34 | 10.10 | NA | 1.98 | NA | NA | NA | NA | NA | NA |
| 35 | NA | NA | NA | NA | NA | NA | 1.28 | NA | 1.30 | NA | NA | NA | NA | NA |
| 36 | NA | NA | NA | NA | 5.72 | 13.48 | 3.21 | 1.95 | NA | NA | NA | NA | NA | NA |
| 37 | NA | NA | NA | NA | NA | NA | 3.51 | 2.32 | NA | NA | NA | NA | NA | NA |
| 38 | NA | NA | 1.93 | 9.69 | NA | NA | NA | 1.78 | NA | NA | NA | NA | NA | NA |
| 39 | NA | NA | NA | NA | NA | NA | 2.22 | NA | NA | NA | NA | NA | NA | NA |
| 40 | NA | NA | NA | NA | 2.47 | 7.14 | NA | NA | NA | NA | NA | NA | NA | NA |
| 41 | NA | NA | NA | NA | 3.71 | 12.81 | 2.98 | 2.79 | 6.79 | NA | NA | NA | NA | NA |
| 42 | NA | NA | NA | NA | 7.81 | 12.55 | 4.90 | NA | 1.14 | NA | NA | NA | NA | NA |
| 43 | NA | NA | NA | NA | NA | NA | 1.83 | 2.24 | 3.04 | NA | NA | NA | NA | NA |
| 44 | NA | NA | NA | NA | NA | NA | 2.08 | NA | NA | NA | NA | NA | NA | NA |
| 45 | NA | NA | NA | NA | NA | NA | 1.83 | NA | NA | NA | NA | NA | NA | NA |
| 46 | NA | NA | NA | NA | 1.95 | 7.15 | 2.71 | NA | 2.27 | NA | NA | NA | NA | NA |
| 47 | NA | NA | NA | NA | NA | NA | 3.59 | 2.57 | NA | NA | NA | NA | NA | NA |
| 48 | NA | NA | NA | NA | NA | 6.01 | 2.05 | 2.46 | 2.14 | NA | NA | NA | NA | NA |
| 49 | NA | NA | 2.68 | 9.72 | NA | NA | NA | NA | NA | NA | NA | NA | NA | NA |
| 50 | NA | NA | NA | NA | NA | NA | NA | 4.28 | NA | NA | NA | NA | NA | NA |
| 51 | NA | NA | NA | NA | NA | 12.87 | 2.94 | 2.14 | 1.36 | NA | NA | NA | NA | NA |
| 52 | NA | NA | NA | NA | 2.06 | 2.71 | 2.01 | 1.38 | 2.40 | NA | NA | NA | NA | NA |
| 53 | NA | NA | NA | NA | 3.25 | 12.62 | NA | 1.76 | 1.95 | NA | NA | NA | NA | NA |
| 54 | 2.88 | 13.94 | 2.80 | 17.16 | NA | NA | NA | 1.86 | NA | NA | NA | NA | NA | NA |
| 55 | NA | NA | NA | NA | 1.48 | 4.91 | 1.78 | NA | 1.34 | NA | NA | NA | NA | NA |
| 56 | NA | NA | NA | NA | NA | NA | 2.86 | NA | NA | NA | NA | NA | NA | NA |
| 57 | NA | NA | NA | NA | NA | 5.22 | 3.90 | 3.29 | 2.85 | NA | NA | NA | NA | NA |
| 58 | NA | NA | 3.19 | 16.19 | NA | NA | 1.80 | 2.86 | 2.31 | NA | NA | NA | NA | NA |
| 59 | NA | NA | NA | NA | NA | NA | 1.39 | NA | 1.41 | NA | NA | NA | NA | NA |
| 60 | NA | NA | NA | NA | NA | NA | NA | 3.35 | NA | NA | NA | NA | NA | NA |
| 61 | NA | NA | NA | NA | 4.52 | NA | 3.55 | 2.78 | NA | NA | NA | NA | NA | NA |
| 62 | NA | NA | NA | NA | NA | NA | 2.53 | 1.25 | NA | NA | NA | NA | NA | NA |
| 63 | NA | NA | NA | NA | NA | NA | 2.10 | 3.84 | NA | NA | NA | NA | NA | NA |
| 64 | NA | NA | NA | NA | 2.38 | 36.18 | 5.01 | 5.03 | 3.18 | NA | NA | NA | NA | NA |
| 65 | NA | NA | 2.56 | 24.33 | NA | NA | 2.17 | 2.28 | 2.57 | NA | NA | NA | NA | NA |
| 66 | NA | NA | NA | NA | NA | NA | 2.86 | NA | NA | NA | NA | NA | NA | NA |
| 67 | NA | NA | NA | NA | 3.69 | 6.46 | NA | NA | NA | NA | NA | NA | NA | NA |
| 68 | NA | NA | NA | NA | 2.04 | 16.29 | 6.53 | 1.88 | 2.40 | 3.28 | 2.10 | 2.76 | 2.66 | 2.90 |
| 69 | 3.12 | 19.62 | NA | NA | NA | NA | NA | 1.52 | 2.03 | NA | NA | NA | NA | NA |
| 70 | NA | NA | NA | NA | 3.68 | 11.71 | 2.41 | 2.54 | 3.64 | NA | NA | NA | NA | NA |
| 71 | NA | NA | NA | NA | 2.60 | 9.01 | 1.98 | NA | 3.25 | NA | NA | NA | NA | NA |
| 72 | NA | NA | NA | NA | NA | NA | 2.08 | NA | NA | NA | NA | NA | NA | NA |
| 73 | NA | NA | NA | NA | 1.67 | 24.33 | 1.64 | 1.30 | 1.28 | NA | NA | NA | NA | NA |
| 74 | NA | NA | NA | NA | 2.76 | 8.73 | 2.95 | NA | NA | NA | NA | NA | NA | NA |
| 75 | NA | NA | NA | NA | 1.33 | 2.99 | 4.83 | NA | 4.65 | NA | NA | NA | NA | NA |
| 76 | NA | NA | NA | NA | 2.74 | 3.73 | 2.22 | NA | NA | NA | NA | NA | NA | NA |
| 77 | NA | NA | NA | NA | NA | NA | 3.93 | 1.33 | NA | 3.92 | 1.85 | 2.69 | 1.54 | 2.49 |
| 78 | NA | NA | NA | NA | NA | NA | 1.78 | NA | NA | NA | NA | NA | NA | NA |
| 79 | NA | NA | 2.60 | 6.99 | NA | NA | 2.23 | 2.40 | NA | NA | NA | NA | NA | NA |
| 80 | 1.29 | 7.50 | NA | NA | NA | NA | NA | NA | NA | NA | NA | NA | NA | NA |
| 81 | NA | NA | NA | NA | NA | NA | NA | 2.13 | NA | NA | NA | NA | NA | NA |
| 82 | NA | NA | NA | NA | NA | NA | 2.75 | NA | NA | NA | NA | NA | NA | NA |
| 83 | NA | NA | NA | NA | 5.85 | 24.67 | 3.01 | 1.54 | 2.32 | NA | NA | NA | NA | NA |
| 84 | NA | NA | NA | NA | NA | NA | 2.74 | 3.20 | NA | NA | NA | NA | NA | NA |
| 85 | NA | NA | 2.52 | 14.05 | NA | NA | 1.73 | 1.65 | 3.20 | NA | NA | NA | NA | NA |
| 86 | NA | NA | NA | NA | NA | NA | NA | 2.28 | NA | NA | NA | NA | NA | NA |
| 87 | NA | NA | NA | NA | 3.13 | NA | 2.89 | 2.17 | 3.51 | NA | NA | NA | NA | NA |
| 88 | NA | NA | NA | NA | NA | NA | NA | NA | 1.23 | NA | NA | NA | NA | NA |
| 89 | NA | NA | NA | NA | 4.04 | 22.37 | 5.30 | 2.26 | NA | NA | NA | NA | NA | NA |
| 90 | NA | NA | NA | NA | 3.05 | 6.04 | NA | NA | 1.42 | NA | NA | NA | NA | NA |
| 91 | NA | NA | NA | NA | 2.13 | 3.15 | NA | NA | 4.30 | NA | NA | NA | NA | NA |
| 92 | NA | NA | NA | NA | NA | NA | NA | 1.97 | NA | NA | NA | NA | NA | NA |
| 93 | NA | NA | NA | NA | NA | NA | NA | 2.24 | NA | NA | NA | NA | NA | NA |
| 94 | NA | NA | NA | NA | NA | NA | NA | 1.99 | NA | NA | NA | NA | NA | NA |
| 95 | NA | NA | NA | NA | 2.28 | NA | NA | 1.66 | 1.50 | NA | NA | NA | NA | NA |
| 96 | NA | NA | NA | NA | 2.42 | 27.61 | NA | 1.48 | NA | NA | NA | NA | NA | NA |
| 97 | NA | NA | NA | NA | NA | 4.20 | NA | NA | 1.64 | NA | NA | NA | NA | NA |
| 98 | NA | NA | NA | NA | 4.30 | 13.54 | 2.48 | 2.18 | 1.78 | NA | NA | NA | NA | NA |
| 99 | NA | NA | NA | NA | NA | NA | 4.85 | NA | NA | NA | NA | NA | NA | NA |
| 100 | NA | NA | NA | NA | NA | NA | 2.47 | NA | NA | NA | NA | NA | NA | NA |
| 101 | NA | NA | NA | NA | NA | NA | 2.19 | 1.27 | 2.03 | 2.90 | 1.55 | 1.50 | 2.20 | 1.60 |

**Table S1.** Table of all salivary cortisol concentrations collected across all timepoints.
