# Supplementary material for: Variation in salivary cortisol responses in yearling Thoroughbred racehorses during their first year of training
Source: PLoS One. 2023 Apr 6;18(4):e0284102. doi: 10.1371/journal.pone.0284102 (PMC10079128; doi:10.1371/journal.pone.0284102)
Supplement: S2 Table — (DOCX) [file pone.0284102.s002.docx]

**Table S2.** Table of statistical details for Anova for timecourse samples.

|  | Df | Sum Sq | Mean Sq | F value | Pr(>F) |  |
| --- | --- | --- | --- | --- | --- | --- |
| time | 4 | 1.01 | 0.25238 | 4.83 | 0.00685 | ** |
| Residuals | 20 | 1.045 | 0.05225 |  |  |  |
