# Supplementary material for: Variation in salivary cortisol responses in yearling Thoroughbred racehorses during their first year of training
Source: PLoS One. 2023 Apr 6;18(4):e0284102. doi: 10.1371/journal.pone.0284102 (PMC10079128; doi:10.1371/journal.pone.0284102)
Supplement: S3 Table — (DOCX) [file pone.0284102.s003.docx]

**Table S3.** Table of P values for Paired t-tests for timecourse samples (df = 4).

| P values | T1 | T2 | T3 | T4 |
| --- | --- | --- | --- | --- |
| T2 | 0.0018 | - | - | - |
| T3 | 0.0167 | 0.3748 | - | - |
| T4 | 0.0252 | 0.9028 | 0.4654 | - |
| T5 | 0.0059 | 0.366 | 0.9113 | 0.4865 |
