# Supplementary material for: Variation in salivary cortisol responses in yearling Thoroughbred racehorses during their first year of training
Source: PLoS One. 2023 Apr 6;18(4):e0284102. doi: 10.1371/journal.pone.0284102 (PMC10079128; doi:10.1371/journal.pone.0284102)
Supplement: S4 Table — (DOCX) [file pone.0284102.s004.docx]

**Table S4.** Table of T values for Paired t-tests for timecourse samples (df = 4).

| T values | T1 | T2 | T3 | T4 |
| --- | --- | --- | --- | --- |
| T2 | -7.36357 | NA | NA | NA |
| T3 | -3.95669 | 0.997948 | NA | NA |
| T4 | -3.48703 | -0.1301 | -0.8061 | NA |
| T5 | -5.34796 | 1.018607 | -0.11863 | 0.765751 |
