# Supplementary material for: Variation in salivary cortisol responses in yearling Thoroughbred racehorses during their first year of training
Source: PLoS One. 2023 Apr 6;18(4):e0284102. doi: 10.1371/journal.pone.0284102 (PMC10079128; doi:10.1371/journal.pone.0284102)
Supplement: S5 Table — (DOCX) [file pone.0284102.s005.docx]

**Table S5.** Table of statistical details for Anova for resting samples.

|  | Df | Sum Sq | Mean Sq | F value | Pr(>F) |
| --- | --- | --- | --- | --- | --- |
| time | 2 | 0.939 | 0.4697 | 2.978 | 0.0569 |
| Residuals | 75 | 11.829 | 0.1577 |  |  |
