# Supplementary material for: Variation in salivary cortisol responses in yearling Thoroughbred racehorses during their first year of training
Source: PLoS One. 2023 Apr 6;18(4):e0284102. doi: 10.1371/journal.pone.0284102 (PMC10079128; doi:10.1371/journal.pone.0284102)
Supplement: S6 Table — (DOCX) [file pone.0284102.s006.docx]

**Table S6.** Table of statistical details for milestone training event samples.

| Milestone event | T value | df | P value |  |
| --- | --- | --- | --- | --- |
| FD | -4.4932 | 5 | 0.00644 | ** |
| FR | -10.481 | 33 | 4.93E-12 | **** |
| FG | -11.219 | 9 | 1.36E-06 | **** |
