# Supplementary material for: Variation in salivary cortisol responses in yearling Thoroughbred racehorses during their first year of training
Source: PLoS One. 2023 Apr 6;18(4):e0284102. doi: 10.1371/journal.pone.0284102 (PMC10079128; doi:10.1371/journal.pone.0284102)
Supplement: Attachment — Submitted filename: Response to Reviewers.docx [file pone.0284102.s007.docx]

**PONE-D-22-23041**

**Consent obtained:**

Verbal consent was obtained from the owner/trainer of the horses, Mr J.S. Bolger, in presence of authors A. Holtby, L.Katz, E.Hill

**Updated competing interests statement:**

This research was carried out with the financial support of Plusvital Ltd. Plusvital is an equine nutrition and genetic testing company in which EWH is a shareholder. This does not alter our adherence to  PLOS ONE policies on sharing data and materials.

**Review Comments to the Author**

**Reviewer #1:** This is a well-described and easy-to-follow paper in which all the experimental procedures were correctly described, the statistical analysis was appropriate (although with some concerns), and the discussion was correctly developed.

However, quite comments have been made by this reviewer related, mainly to the discussion results.
Please answer the following questions in detail:

| **Reviewer Comments** | **Author responses** |
| --- | --- |
| Lines 23-24: Response to Acute stress is not solely produced by the HPA but also by the autonomic nervous via the sympathetic system and Sympathetic adrenal medullary, which are faster in acting after an acute stressor and are responsible for that fight-or-flight response. | Thank you for pointing this out, we completely agree. We have rephrased to clarify. |
| Lines 29-34: This is not the study's main objective. Therefore, it could be better to first describe the results from the other experiment (evaluate the acute stress by the pre- and post-novel training events) and then describe this. Since this first experiment only was performed, if this reviewer understood correctly, to check what would be the basal salivary cortisol levels at resting and if daily variations or other external environment variations could bias the salivary cortisol results for the main experimental activity of this study: if variations in horses' salivary cortisol can explain horses' variations due to their temperaments after backing by a jockey for the first time. | We understand this comment, and appreciate the suggestion. The purpose for describing these results ahead of the pre- and post- novel training event sample results is to provide the background framework, and give further context around all samples collected from the horses used in the study. |
| Line 44 (Keywords): This reviewer advises including the word "saliva". | Thank you for this suggestion, we have added “saliva” as a keyword. |
| Line 161: When authors explain horses did not receive hard feed, did it mean neither hay for two hours? This reviewer thinks to avoid feed contamination in the saliva sample recollections, isn't it? Salivary contamination by feed can influence salivary cortisol concentrations [1]. | Yes, that is correct; salivary contamination by feed can affect salivary cortisol measurements. The horses in this study did not have any hard food / ‘concentrate’ i.e. oats / commercial food mix / chaff for the two hours prior to sample collection. |
| Lines 232-233: If distribution data behaved as non-parametric distribution, ANOVA and t-test would not be the better to perform the statistical tests unless the authors previously transformed the data to make them normal. | Thank you for noting this. We tested the data for normality and the data has been normalised using a log transformation. All analyses have been repeated using the log transformed data. This has been revised in the text and the details of the test statistics and degrees of freedom have been included as suggested. |
| Lines 348-356: Although not the main objective of this study, this pre-experiment was performed in females, not in males or geldings. However, it is well known that this daily cortisol behaviour is in both sexes. However, this must be figurated in the limitation section. | Yes, we agree. We have added additional detail at line 444 to clarify this. The data collection for this research was convenient sampling and so it was not possible to request additional, male horses for measurement. |
| Lines 397-398: This could be better if, during this study, the authors had measured the behaviour to evaluate the different temperaments of each horse and linked it with the cortisol concentrations. This must be pointed out in the discussion as a possible cause of cortisol variability after an acute stressor. | We agree, we have performed this measurement and the analysis / results are presented in another manuscript currently under review.  We have added a line at 397- to highlight this in the discussion. |
| Lines 451-454: This is not a piece of new evidence (the results from the present study are not a novelty in this aspect). Moreover, of course, the authors have observed individual variations in cortisol concentrations after acute stress. However, to objectively demonstrate the individual variation in the stress response of horses by measuring salivary cortisol and, therefore, try to use salivary cortisol to "identify variation within the Thoroughbred population and indicate vulnerability or strengths in adjusting to the rigours of the racing training environment"; these results must be linked with behaviour response from them and, therefore, with their temperament. | Thank you for noting this, we agree. We carried out behavioural phenotyping and the results of this experiment are presented in another manuscript currently under review We have rephrased these lines to clarify, hopefully this is more clear. |

REFERENCES
1. Contreras-Aguilar MD, Luisa M, Escribano D, Lamy E, Tecles F, Cerón JJ. Effect of food contamination and collection material in the measurement of biomarkers in saliva of horses. Res Vet Sci. 2020;129: 90–95. doi:10.1016/j.rvsc.2020.01.006

Reviewer #2: Congratulations for a nice study on young horses' stress response during their early learning experiences. The study is sound, even if in some places has a limited sample size; the methods regarding salivary sampling or hormone assay are fine, and the results are reported clearly.
I have only a few minor comments, mainly editorial.

| **Reviewer Comments** | **Author responses** |
| --- | --- |
| - l.393 ff. In addition to the cited papers, there's a new one out there that reports no elevated stress response to early training. Although their paper examines training tasks that have limited overlap with yours, it might be worth considering. Their finding is basically that early training does not elevate cortisol, except when training is physically demanding enough to cause the elevation. Niittynen et al, 2022 App. Anim. Behav. Sci. | Thank you for pointing out this new published study, we have added a consideration of the suggested publication to our discussion. |
| - l. 271, 295, 310 and elsewhere: where you report the p-values, please also always report the test statistic and df. | Thank you for this suggestion. The text has been revised as requested. |
| - Figure legend 3. The text indicates that there's significance indicators in the figure, but there are none. | Thank you for pointing out this oversight. This has been amended to feature the significance indicators. |
| - I think some of the tables / figures are somewhat unnecessary. I would leave out altogether Table 3 and Figure 2, and move to the Supplement Figures 4, 5 and 6. |  |
